# Gaps between college and starting an MD-PhD program are adding years to physician-scientist training time

**DOI:** 10.1172/jci.insight.156168

**Published:** 2022-03-22

**Authors:** Lawrence F. Brass, Reiko Maki Fitzsimonds, Myles H. Akabas

**Affiliations:** 1 Department of Medicine, Department of Systems Pharmacology and Translational Therapeutics, and MD-PhD program, University of Pennsylvania Perelman School of Medicine, Philadelphia, Pennsylvania, USA.; 2 Department of Cellular and Molecular Physiology and MD-PhD program, Yale University School of Medicine, New Haven, Connecticut, USA.; 3 Departments of Physiology and Biophysics, Neuroscience, and Medicine and MD-PhD program, Albert Einstein College of Medicine, Bronx, New York, USA.

**Keywords:** Aging, Complex traits

## Abstract

The average age when physician-scientists begin their career has been rising. Here, we focused on one contributor to this change: the increasingly common decision by candidates to postpone applying to MD-PhD programs until after college. This creates a time gap between college and medical school. Data were obtained from 3544 trainees in 73 programs, 72 program directors, and AAMC databases. From 2013 to 2020, the prevalence of gaps rose from 53% to 75%, with the time usually spent doing research. Gap prevalence for MD students also increased but not to the same extent and for different reasons. Differences by gender, underrepresented status, and program size were minimal. Most candidates who took a gap did so because they believed it would improve their chances of admission, but gaps were as common among those not accepted to MD-PhD programs as among those who were. Many program directors preferred candidates with gaps, believing without evidence that gaps reflects greater commitment. Although candidates with gaps were more likely to have a publication at the time of admission, gaps were not associated with a shorter time to degree nor have they been shown to improve outcomes. Together, these observations raise concerns that, by promoting gaps after college, current admissions practices have had unintended consequences without commensurate advantages.

## Graphical abstract

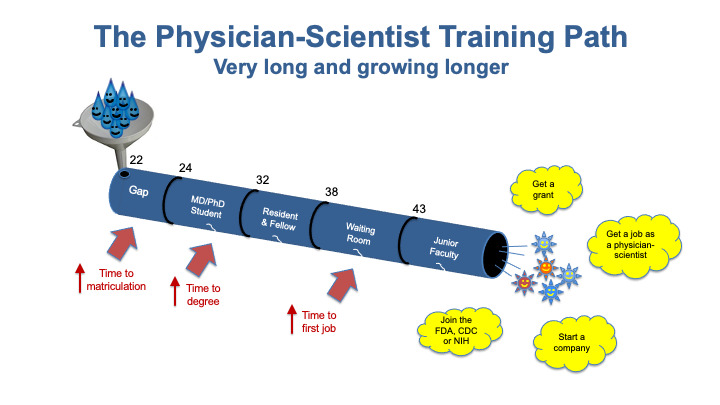

## Introduction

Integrated MD-PhD programs were developed in the 1950s to provide rigorous research training to future physician-scientists that is not part of the standard medical school curriculum (1, 2). The earliest programs were few in number and limited in both size and diversity. Candidates to those programs typically applied after their junior year in college and began training within a few months of graduating from college. Since the 1950s, the national need for more physician-scientists and the contribution of MD-PhD programs to meeting that need has been recognized (3–5). As a result, substantial NIH and institutional resources have been invested to increase the number of programs, the number of trainees per program, and the diversity of students enrolled (3, 6, 7). At present, there are approximately 95 active programs, which we defined here as having at least 10 total trainees in the academic year ending in 2021 (AY2021). Fifty of the 95 programs were receiving National Institute of General Medical Sciences (NIGMS) training support in the form of Medical Scientist Training Program (MSTP) T32 grants when this manuscript was being written (8). Nearly 6000 students are currently enrolled (9), and 600–700 new students begin training each year (2, 10).

At the time that MD-PhD programs were expanding, there was considerable discussion about the limited number of undergraduates pursuing careers as physician-scientists (3, 11–13). Several factors that dissuaded applicants were identified, including the extended training time, the increasing age at which physician-scientists establish independent research careers, the opportunity costs of extended training time, concerns about work/life imbalance, competition for faculty appointments with protected research time, and the challenges of sustaining a research career over many years (3, 14). Total training time traditionally includes the time to degree in MD-PhD programs plus the time to complete postgraduate training. The duration of both has increased over the past few decades, such that trainees may not achieve an independent research position until they are in their late 30s or early 40s (2, 13, 15).

Here, we have focused on an underappreciated factor that increases the average age at which physician-scientists launch their independent careers: the growing tendency for undergraduates to postpone applying to MD-PhD programs until after they have finished college, rather than before their senior year. In 2020, two of the authors of the present study invited members of the National Association of MD-PhD Programs to participate in a pilot project to test their anecdotal impression that most students entering MD-PhD programs had waited to apply until after college. Twenty-two programs provided data that suggested that most entering students had done so. Based on that pilot, the present study was launched in early 2021, with invitations to participate sent to the directors of US MD-PhD programs. Most of the directors chose to participate, collectively representing 86% of the 5830 MD-PhD students enrolled in AY2021 and 49 of the 50 programs supported by NIGMS MSTP T32 grants (9). Participating programs were provided a school-specific link to an anonymous online survey to forward to their trainees. Participating trainees were asked when they graduated from college and when they started medical school. If there was a gap, they were asked why they had waited to apply, how they had used the time, from whom they had obtained advice, and whether they would recommend their choice to others. The survey also asked about their undergraduate research experience and publication record at the time that they applied to MD-PhD programs. A survey sent to directors asked whether their program’s application requested information about gaps and publications and, if they did, how they used this information in making decisions. Finally, we asked program directors for time-to-degree and gap length data for their recent graduates.

The surveys were completed by 3544 trainees (71%) and all but 1 program director. The results were combined with data on all applicants and matriculants obtained from the Association of American Medical Colleges (AAMC) Matriculant Data File. The results of the trainee survey show that gap prevalence among successful MD-PhD program applicants has steadily increased over the past decade. Although other reasons were identified, the increase reflects a common impression among applicants that a gap spent doing research is necessary for a successful application. The directors’ survey showed that many programs do pay attention to whether an applicant had devoted time after graduation to research but not to the extent that many applicants believe. Notably, we found no evidence in either the literature or this study that gaps shorten the time to degree or improve performance during or after graduation. This does not mean that gaps have no value to individual applicants, just that they should not be viewed as a requirement for admission to an MD-PhD programs.

## Results

Online surveys were distributed to students in US MD-PhD programs and their program directors in early 2021. Copies of each survey are included in the Supplemental Methods (supplemental material available online with this article; https://doi.org/10.1172/jci.insight.156168DS1 The overall survey participation rate for MD-PhD program trainees was 71% (3544 of 5007 trainees in 73 programs, Supplemental Table 1). Average participation rates were slightly lower for large programs (≥100 students; 67% participation rate) than for small programs (<60 students; 75%) and for those programs with NIGMS MSTP T32 grants (70%) compared with those without (76%). 54% of participants identified as male, 44% as female, 0.8% as nonbinary, and 0.5% declined to answer; 15% identified themselves as members of one or more groups considered to be underrepresented in medicine (UIM, defined in Methods section). Results from the trainee survey were compared with data from the annual AAMC Matriculating Student Questionnaire (MSQ), which includes questions about time spent between college and medical school. Although the MSQ doesn’t separate answers from MD and MD-PhD students, it predominantly reflects MD candidates, who are approximately 97% of US medical school matriculants.

### Gap prevalence has increased substantially.

**Figure 1:**
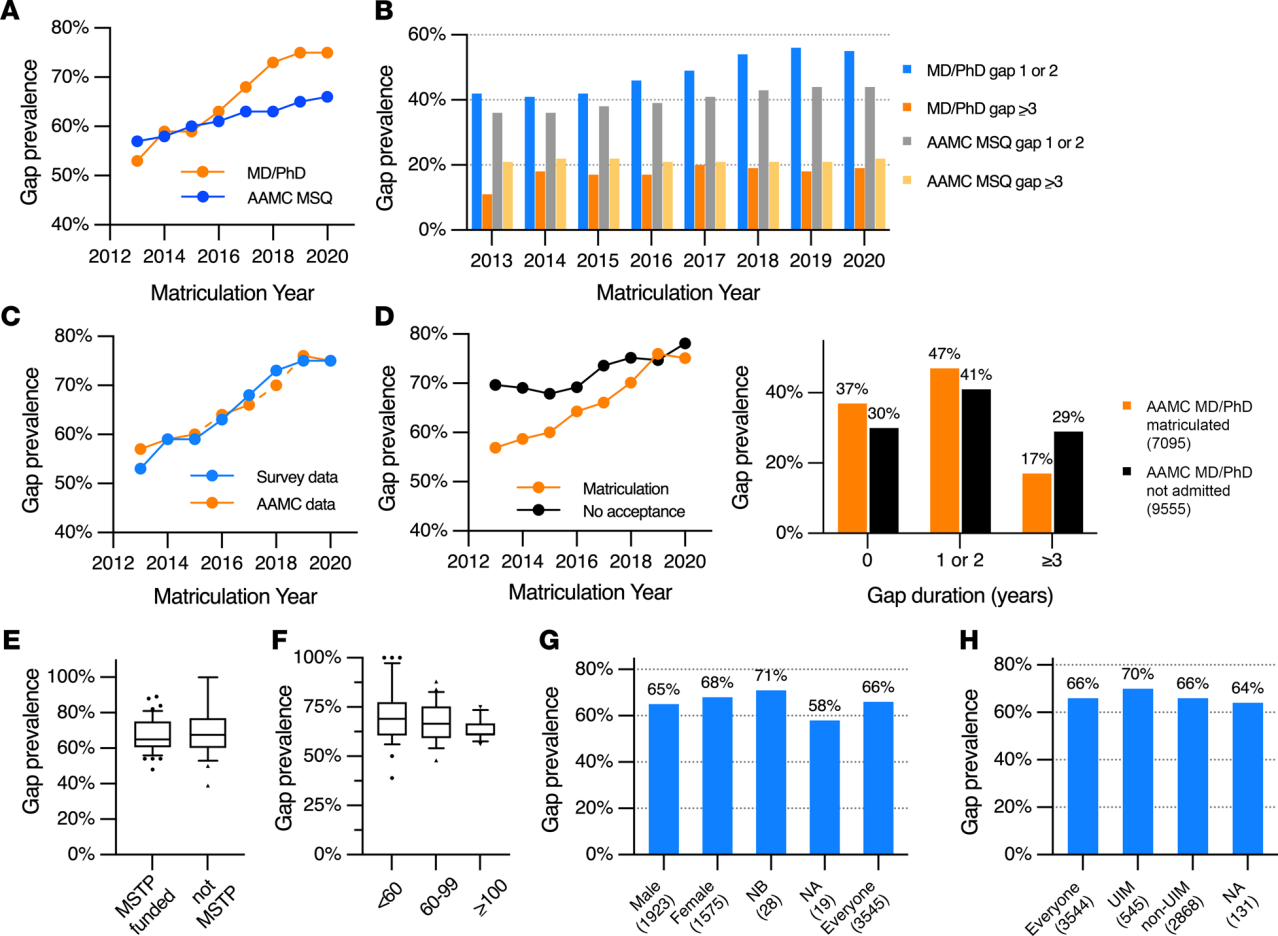
Gaps between college and MD-PhD program matriculation. (**A**) Gap prevalence by matriculation year. Data on matriculating medical students (MD and MD-PhD) were obtained from the AAMC MSQ (*n* = 12,779–16,668). MD-PhD data from current survey respondents (matriculation year 2013–2020, *n* = 306, 355, 391, 419, 424, 472, 451, and 519, respectively). (**B**) Shorter versus longer gaps by matriculation year. (**C**) Comparison of gap prevalence for current MD-PhD matriculants derived from present survey data with gap prevalence derived from AAMC data on all MD-PhD program matriculants from 2013–2020 (*n* = 605–707/year, 5223 total). (**D**) Comparison of AAMC gap prevalence data for MD-PhD matriculants from 2013 to 2020 (*n* = 605–707/ year, 5223 total) with gap prevalence data for those who were not accepted (*n* = 957–1064/year, 8007 total). Comparison of gap duration for MD-PhD matriculants compared with those who were not admitted. (**E**) Average gap prevalence for trainees in NIGMS MSTP training grant-supported programs (*n* = 49 programs, *n* = 3068 respondents, 70% participation rate) versus trainees from programs without MSTP grants (*n* = 25 programs, *n* = 474 respondents, 76% participation rate) in 2021. Mean + SD. Boxes indicate the 25th to 75th percentiles; lines within the boxes indicate medians, and whiskers indicate the 10th and 90th percentiles. Points outside of whiskers are shown. Differences are not statistically significant by ANOVA with Tukey’s HSD post hoc test. (**F**) Programs were grouped by those with fewer than 60 trainees (group 1, *n* = 33 programs, *n* = 803 respondents, 75% participation rate), programs with 60–99 trainees (group 2, *n* = 26 programs, *n* = 1482 respondents, 72% participation rate), and programs with 100 or more trainees (group 3, *n* = 14 programs, *n* = 1257 respondents, 67% participation rate). Mean + SD. Differences are not statistically significant by 1-way ANOVA. (**G**) Gap prevalence by gender. NB, nonbinary; NA, declined to answer. (**H**) Gap prevalence by race/ethnicity. NA, declined to answer. (**G** and **H**) Parenthetical number represents total number of respondents in each group.

Herein, we define a “gap” as the time between college graduation and matriculation into an MD-PhD program, with 0 years meaning that the student went straight from college into MD-PhD training with no time in between. Figure 1 illustrates gap prevalence, defined as the percentage of matriculating students who took a gap of ≥1 year, as a function of entry year from 2013 to 2020. The data show that gaps have become more prevalent for medical students in general during this period, but they are even more so for trainees in MD-PhD programs, rising from 53% among those who entered in 2013 to 75% for those who entered in 2020 (Figure 1A). Most of the upward trend is observed in those trainees who had a 1- or 2-year gap and not those whose gap lasted 3 or more years (Figure 1B).

Because the survey data covered only 71% of current trainees, we also obtained deidentified AAMC data for all applicants to MD-PhD programs from 2013 to 2020. The data included the year applicants graduated from college, whether they matriculated into an MD-PhD program and the year they entered, and whether they were accepted somewhere or rejected everywhere. Figure 1C compares gap prevalence in the AAMC data set with data obtained in the present survey. The two curves are essentially superimposable, which suggests that there was little if any difference between those who responded to the present survey and those who did not with respect to gap prevalence. Figure 1D compares gap prevalence for those who matriculated into an MD-PhD program with those whose applications were unsuccessful. If anything, gap prevalence was greater among those who were not accepted than those who were, although this difference has gradually declined (Figure 1D, left). When grouped by gap duration, unsuccessful applicants were more likely to have taken longer gaps (Figure 1D, right).

The average gap prevalence by program for MD-PhD students varied considerably, ranging from 39% to 100%. There was no significant difference between programs currently funded by NIGMS MSTP T32 grants and those that are not, although the range of gap prevalence was wider in the programs that did not have NIGMS T32 funding (39%–100% vs. 48%–89%) (Figure 1E). There was also little difference by program size, gender, or self-identification as a member of a group considered to be UIM (Figure 1, F–H).

### Gap length distribution.

**Figure 2:**
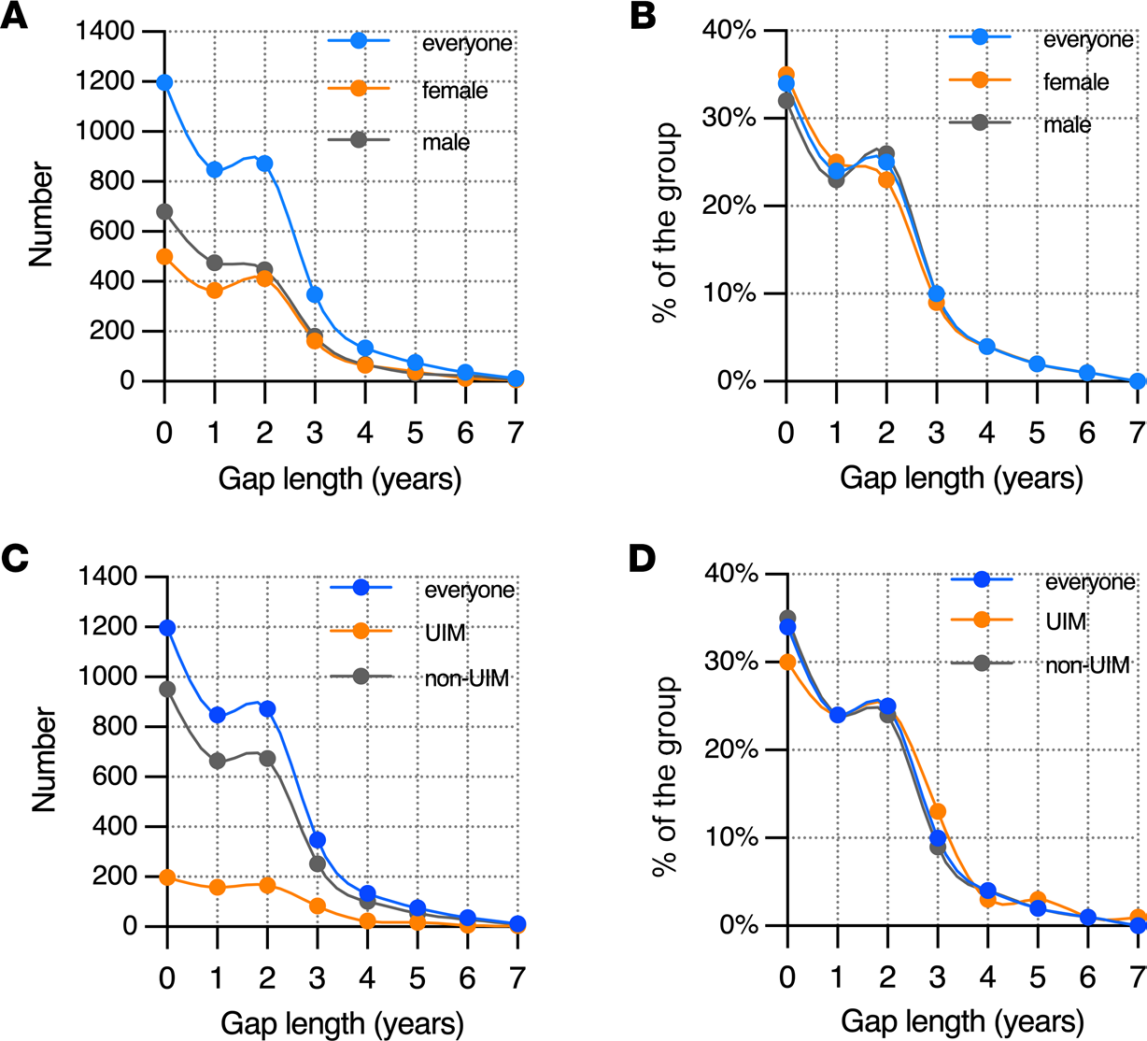
Gap length distribution by gender and by race and ethnicity status. (**A** and **C**) Total number of survey respondents in each group who had a gap of the indicated duration following college graduation. (**B** and **D**) Percentage of each group who took a gap of the indicated duration. Total number of survey respondents in each group is indicated in Figure 1, G and H.

The data in Figure 2 show the length of the gaps taken by current trainees who answered the survey. Among those who took a gap, durations of 1 or 2 years were by far the most common. Gaps lasting 3 or more years were reported by 629 of 3544 trainees (18%). There were no meaningful differences in gap length distribution by gender or among those who identified as belonging to groups considered to be UIM.

### Prior research experience.

**Figure 3:**
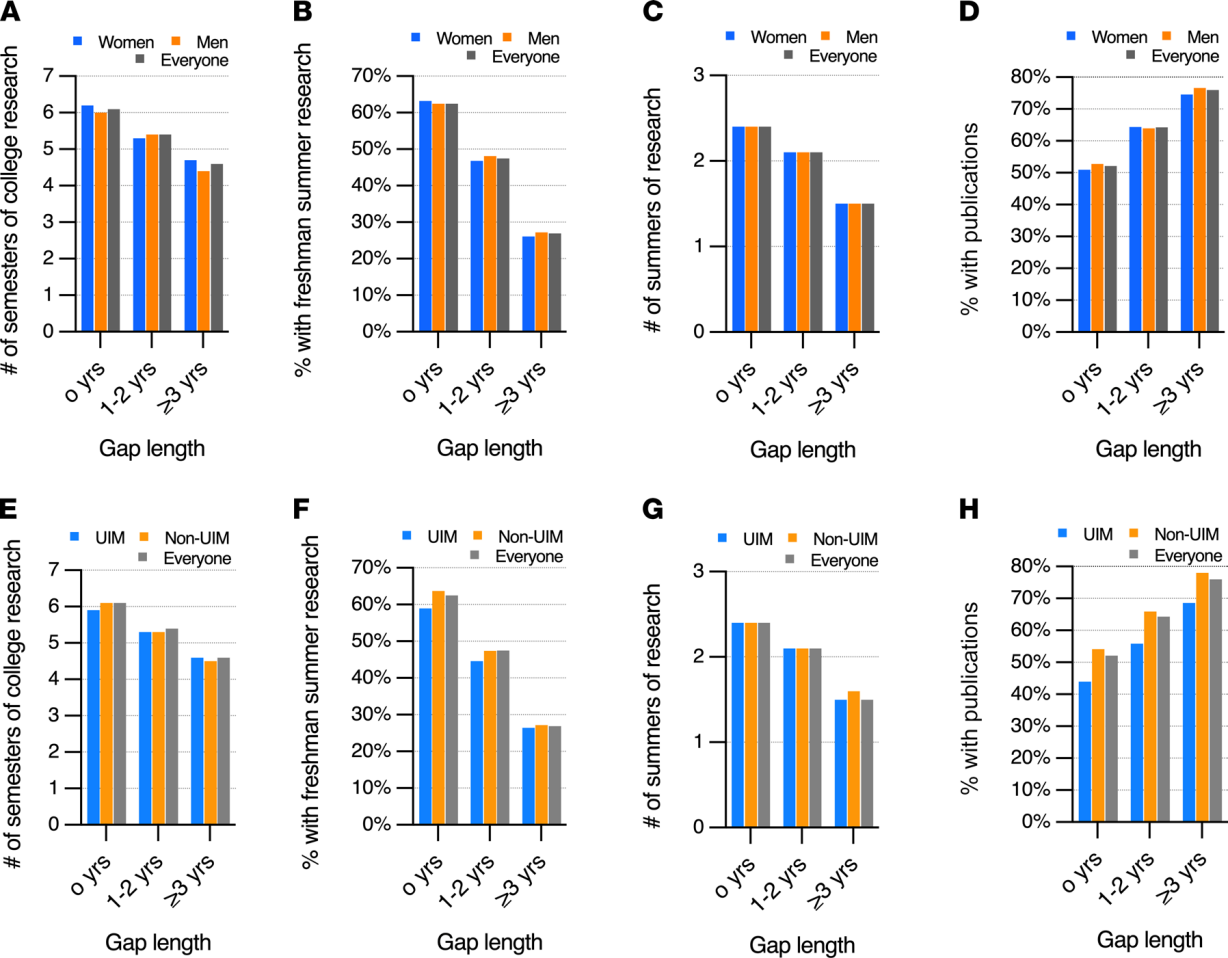
Extent of research experiences during college and likelihood of having publications at the time of MD-PhD application by gap duration. Survey respondents were divided into those with no gap (*n* = 1196), a 1- to 2-year gap (*n* = 1719), and a gap of ≥3 years (*n* = 629). (**A–D**) Comparison of women (*n* = 1574) and men (*n* = 1923). (**E–H**) Comparison of those from groups considered to be underrepresented in medicine (UIM) (*n* = 545) to those who are not (non-UIM) (*n* = 2868). (**A** and **E**) Average number of semesters of research during college. (**B** and **F**) Percentage of those surveyed who reported a summer research experience between freshman and sophomore years. (**C** and **G**) Average number of summers of research. (**D** and **H**) Percentage of those surveyed who reported having a publication at the time of submission of their MD-PhD program application.

Figure 3 summarizes data on research experience and publications at the time of application. In general, students who had more research time in college (semesters and summers) were less likely to take a gap (Figure 3, A and E). Those who chose to not take a gap were more likely to have done research during the summer after their freshman year (Figure 3, B and F) and to have more total summers of research (Figure 3, C and G). Overall, 2205 of 3544 survey respondents (62%) reported having a publication at the time of application, with the likelihood of having a publication increasing among those with longer gaps (Figure 3, D and H). Each of these differences remained consistent across comparisons by gender and membership in groups considered to be UIM.

### Undergraduate major and the decision to do a gap.

**Figure 4:**
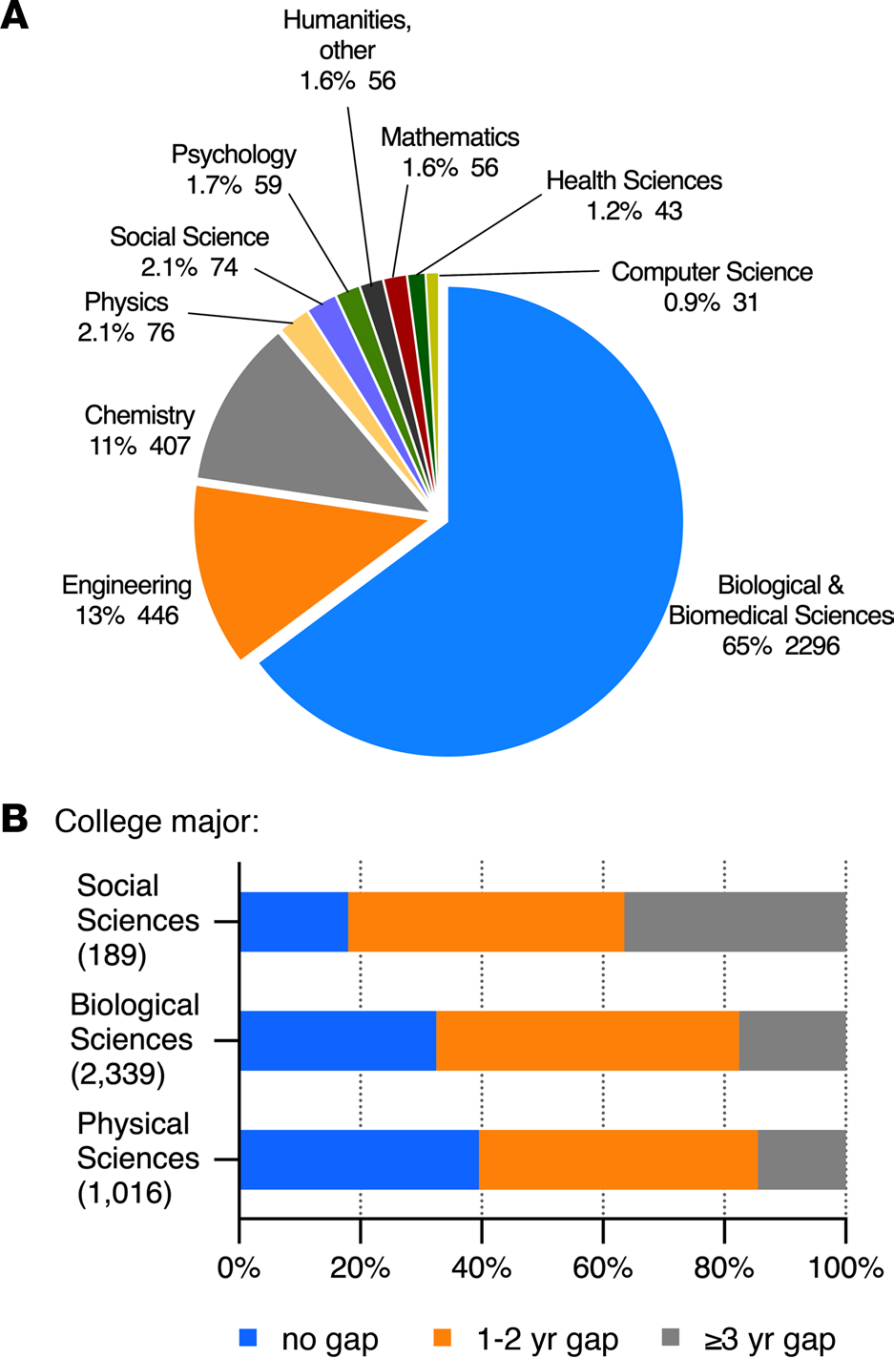
Distribution of undergraduate majors among MD-PhD students and gap length as a function of category of undergraduate major. (**A**) Percentage of students reporting an indicated undergraduate major from a dropdown list of majors. Number of respondents and percentage of total (*n* = 3544). (**B**) Respondent’s majors were grouped into three categories: social sciences (includes humanities, social sciences, and psychology), biological sciences, and physical sciences (includes chemistry, computer sciences, engineering, mathematics, and physics). The percentage of students in a given category who had no gap (blue), a 1- to 2-year gap (orange), or a gap of 3 or more years (gray) is shown. The total number of students in each category is shown in parentheses. Note that the number of students with majors in the social sciences is much smaller than in the other two categories.

Most current trainees in MD-PhD programs who responded to the survey indicated that their undergraduate degrees were in the biological sciences. Smaller numbers majored in the physical sciences (Figure 4A). The smallest group consisted of those who were social science majors in college. Gap prevalence was greatest (82%) among the social sciences majors, who also tended toward longer gaps. Those who had been physical science majors were least likely to have a gap (61%) (Figure 4B).

### Why did you do a gap?

A large part of the trainee survey focused on the decision to do a gap. Trainees who had taken a gap were asked to select from a predefined list of possible reasons. Their choices are summarized in Table 1. They were also given the opportunity to select “other” and write in additional reasons (Supplemental Table 2). Multiple selections were allowed, but in a subsequent question they were also asked to identify which of their selections they considered to be the primary reason. The most frequently selected reasons were “I thought that more research experience would make me a more competitive applicant” (66%) and “I wanted more research experience to solidify my decision to pursue a research-active career” (60%). Burnout, learning about MD-PhD programs too late, wanting more clinical experience, and needing to make money were each selected by about one-quarter of respondents. Needing the time to repeat the MCAT, complete prerequisites, and wanting to improve their academic record were selected least frequently. Of the 487 (14%) who selected “other,” the most common reasons were work and fellowship opportunities, not being sure about whether to apply, and needing to reapply. For the most part, there were few differences between men and women, between those who self-identified as UIM and those who did not, and between those whose gap lasted for 1 or 2 years and those whose gap was ≥3 years. However, individuals from groups considered to be UIM were more likely to list the need to make money or repeat the MCAT and were less likely to want to take personal time. Women were more likely than men to say that they were burnt out from school and needed time off. Those who took longer gaps were more likely to list wanting more research experience, more clinical experience, needing to make money, needing to retake the MCAT, improving grades, and prerequisite courses (Table 1).

**Figure 5:**
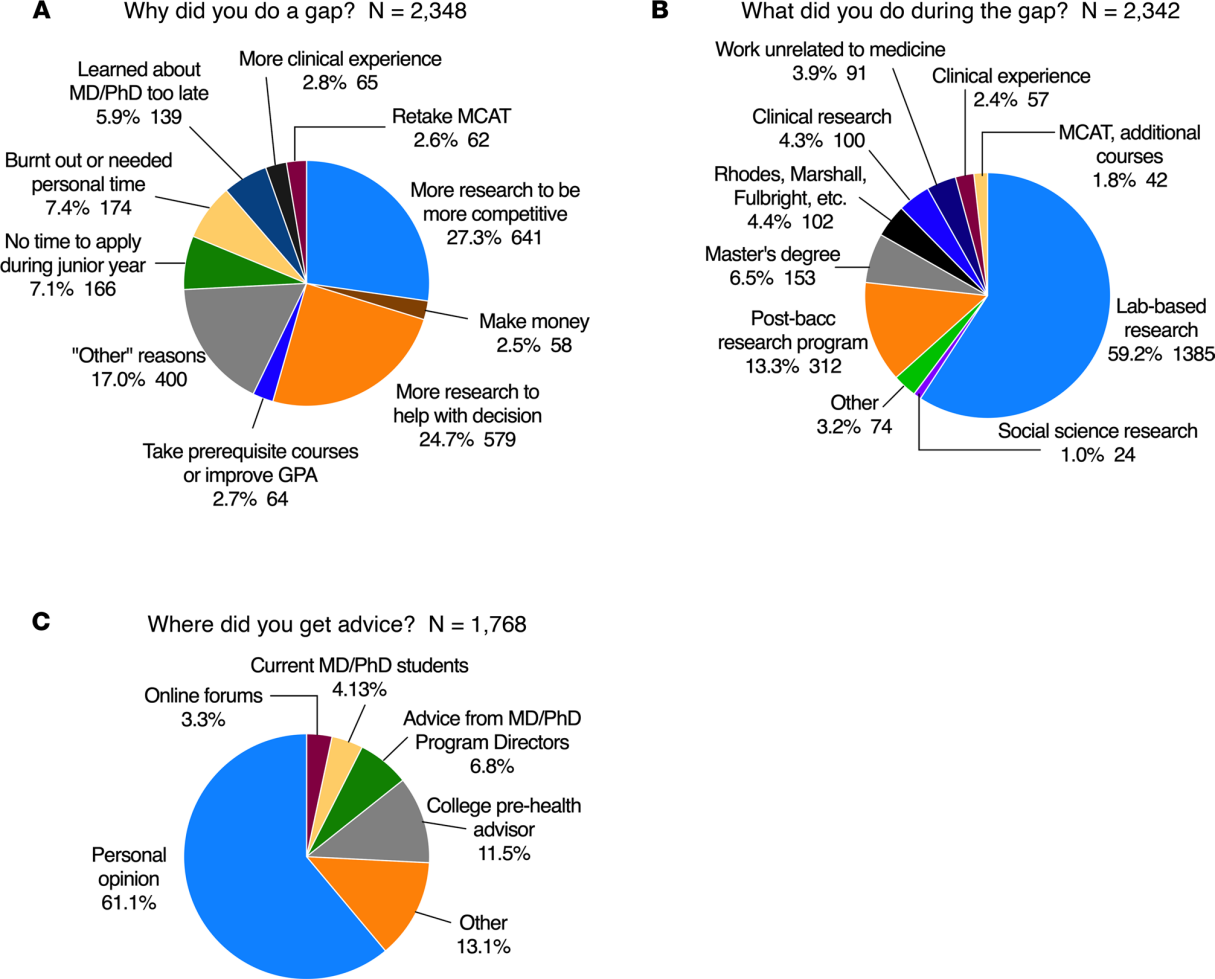
MD-PhD program trainees’ primary reasons for taking a gap, primary activity during the gap, and primary source of advice. The students who had taken a gap were asked for (**A**) their primary reasons for doing a gap, (**B**) what they did during it, and (**C**) from where their advice came. Their choices and the percentage of respondents who selected that choice are shown on the pie charts. See also Tables 1, 3, and 6 for all of the reasons that were listed.

**Figure 6:**
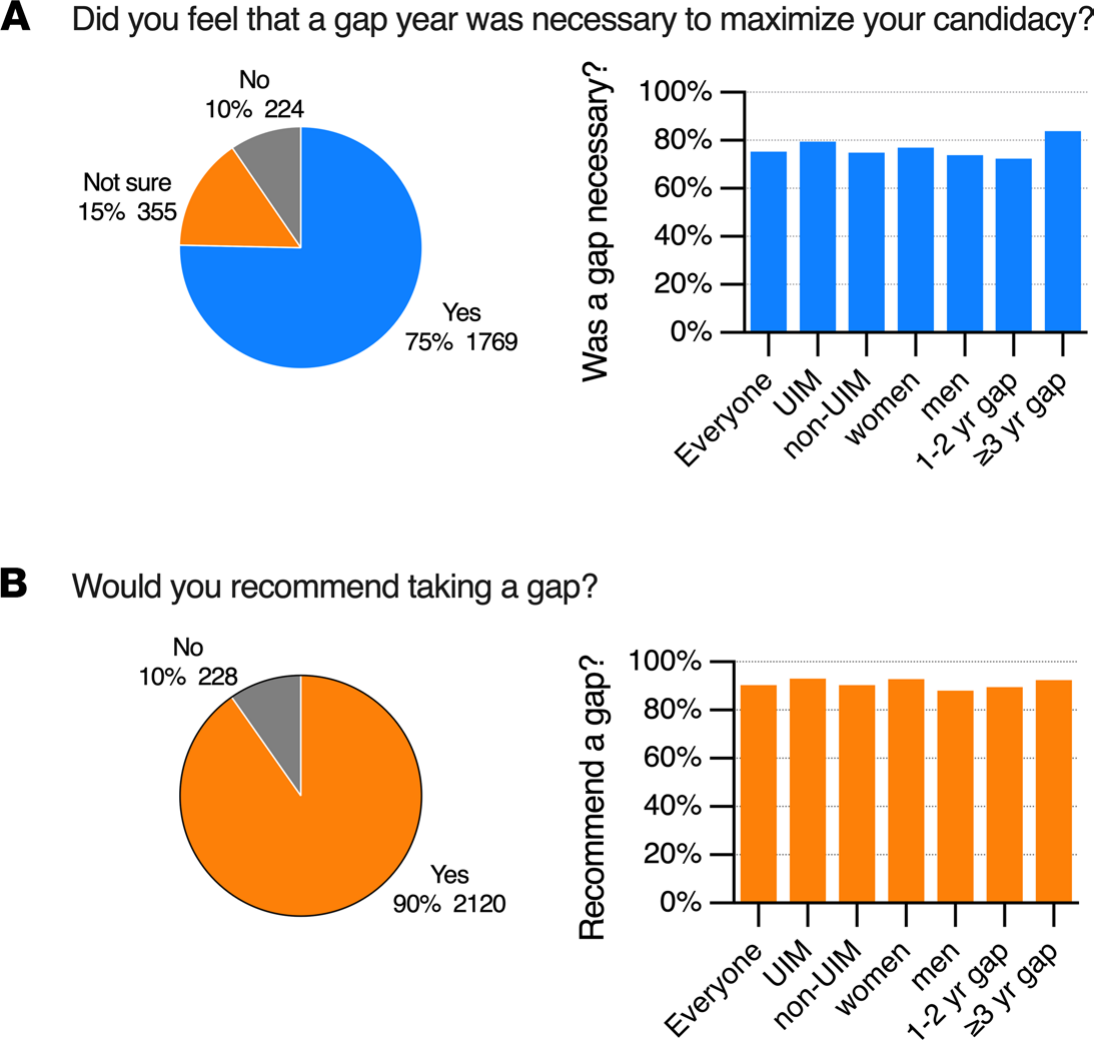
Trainee views on the necessity and advisability of taking a gap. For the students who took a gap, responses to whether they felt a gap was necessary to maximize their candidacy and whether they would recommend taking a gap to future applicants. Number of respondents and the percentage of the total respondents for each response is shown. (**A**) The percentage who responded that a gap was necessary broken out by UIM status, gender, and gap duration. (**B**) The percentage of respondents who would recommend a gap by UIM status, gender, and gap duration.

When asked to select the primary reason that they had chosen to take a gap, 52% of respondents listed a desire for more research experience, either to make them more competitive or to solidify their decision (Table 2 and Figure 5A). Notably, 75% of those who took a gap after college said that they did so to maximize their candidacy (Figure 6A). Only 10% thought it would not. Nearly everyone who had taken a gap said they would recommend taking one to others (Figure 6B). There was little or no difference in this response between men and women, and between those who self-identified as UIM and those who did not. Those who were most certain about the necessity of time between college and MD-PhD training were those with gaps that lasted 3 or more years; however, these differences were small (Figure 6B).

### What did you do during your time between college and medical school?

Trainees who had taken a gap were asked to select from a predefined list of possible activities during a gap (Table 3). Multiple selections were allowed, but we also asked for what they considered to be the primary reason (Table 4 and Figure 5B). The two most frequently selected activities were “worked/volunteered in a research laboratory” (selected by 80% in Table 3) and “studied for and took the MCAT” (48%). 93% of those with a gap of 1 or 2 years and 97% of those taking a longer gap reported being in paid positions. We did not ask whether the pay that the received was sufficient for their living costs or whether they required additional support from other sources, including parents.

Taking the MCAT was especially prevalent among those who took gaps of ≥3 years, 81% of whom listed that as one of their activities. Members of groups considered to be UIM were more likely to list additional coursework and postbaccalaureate research programs. Those with longer gaps were more likely to list additional coursework, work in a field unrelated to medicine, and enrollment in a master’s program. “Other” activities included clinical experiences, but the number of people who selected “other” in Table 3 was small. Among the primary activities listed in Table 4 and Figure 5B, working in a research laboratory was by far the most frequent choice (59%), followed by enrollment in a postbaccalaureate research program (13% overall, 21% for individuals from underrepresented groups). Taken together, this indicates that 72% of those who took a gap were doing research as their primary activity.

We compared the responses to our survey with results from the AAMC MSQ survey, where the three most frequently selected activities were “worked in another career” (49% in 2020), “worked/volunteered in research” (48%), and “worked to improve finances” (40%) (Table 5). The MSQ allowed multiple selections; however, the distribution of reasons to do a gap highlights a striking difference between MD-PhD program trainees and medical students in general.

### How did you conclude that a gap was necessary?

By far the most frequent selection was “personal opinion,” a statement that presumably reflects multiple inputs as well as the applicant’s own musings. Other predefined choices in descending frequency were college pre-health advisors, online forums, current MD-PhD students, and advice from MD-PhD program directors (Table 6). The ranked choices that respondents considered to be most influential are shown in Table 7 and Figure 5C. Among the analyzed subgroups, members of underrepresented groups were more likely to acknowledge advice from program directors, as were men. Women were more likely to include college prehealth advisors and men were more likely to mention online forums. Current trainees who had gaps lasting ≥3 years were less likely to cite advice from college prehealth advisors and current MD-PhD students.

Approximately 71% (2511 trainees) of the 3544 trainees who completed the survey identified their undergraduate school. Assuming the survey respondents are representative of the overall MD-PhD trainee population, most current MD-PhD trainees came from a limited number of colleges. Although there were 385 colleges in all, 288 (75%) were represented in the survey by 5 or fewer respondents. Ranked by number of respondents, the top 30 colleges by respondent number (7.8% of 385) supplied 50% of the survey respondents. In this group of colleges, gap prevalence ranged from 15% to 89% (Supplemental Figure 1). One caveat to the data on undergraduate institution is that 29% of respondents did not provide this information. Trainees who attended small colleges may have felt that identifying their college would make their responses identifiable to their program director and thus may have declined to provide the information.

### Results from the survey of program directors.

**Figure 7:**
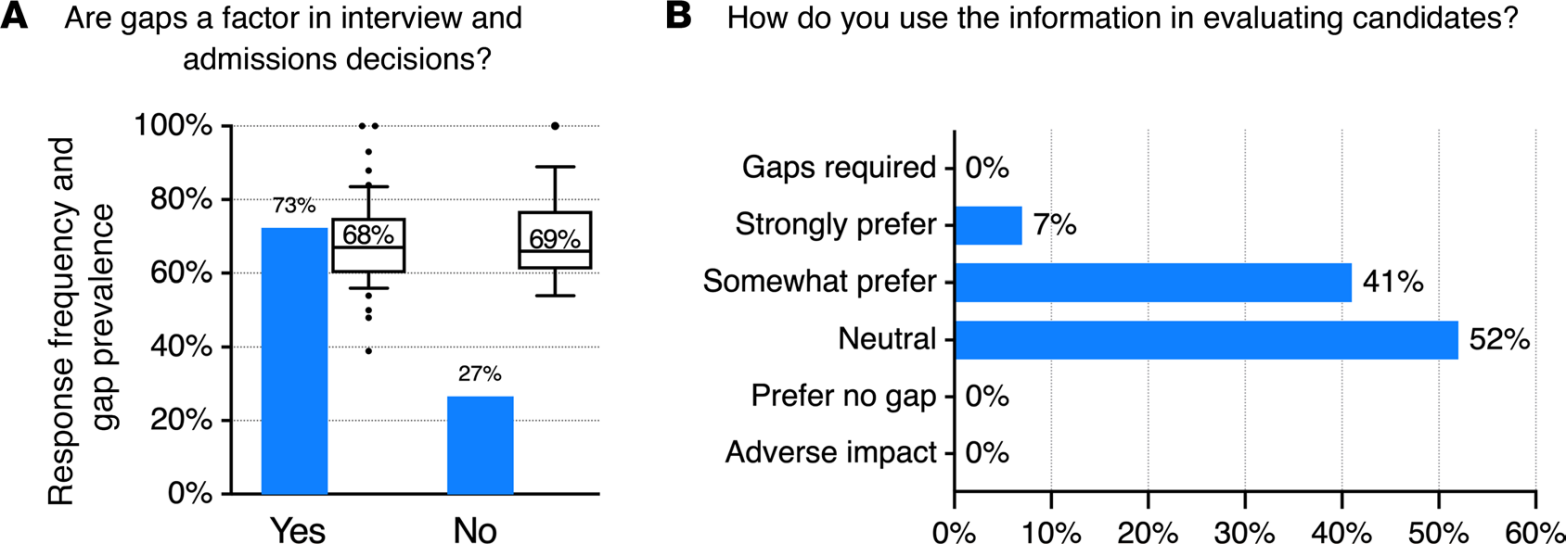
Program directors’ survey. (**A**) Responses to the following question in the program director’s survey: “Are gaps a factor when deciding whom to interview and admit?” Blue bars show the percentage of directors who responded yes or no. The box-and-whisker plots to the right of each blue bar show the gap prevalence in the programs whose directors answered yes or no. Boxes indicate the 25th to 75th percentiles; lines within the boxes indicate medians, and whiskers indicate the 10th and 90th percentiles. Points above and below the whiskers are shown. Numbers indicate average gap prevalence for the programs (*n* = 70). (**B**) Directors who responded that gaps were a factor were asked to indicate the impact on decision making from a dropdown list of responses. The possible choices are shown, with the percentage of respondents choosing a given response (*n* = 51).

After trainee survey collection was complete, a brief survey on related issues was sent to the directors of the 73 participating MD-PhD program, nearly all of whom completed it. One purpose of the survey was to ascertain whether trainees’ impressions that significant research experiences were necessary aligned with the preferences articulated by the program directors. The first question asked whether or not gaps are a factor that programs considered when making interview and admission decisions. 19 of 70 program directors (29%) responded “no” (Figure 7A). Notably, average gap prevalence among trainees who answered the survey was the same in programs that consider gaps a decision factor (68%) as in those that do not (69%).

The 51 program directors who responded that they did take gaps into consideration for interview and admission decisions were then asked, “Which of the following descriptors best reflects how your admissions committee uses that information when evaluating a candidate for interview or acceptance. Assume that the time is spent doing research.” No one indicated a preference for candidates who had just graduated from college or advised candidates to not take a gap (Figure 7B). Conversely, none of the program directors indicated they require a research-focused gap, and only 5 (7%) said they strongly prefer it when making decisions. 46 said that they either somewhat prefer candidates who have taken a gap (29 program directors) or that the presence of a gap has no effect on their decisions (17 program directors). Counting the 21 programs that indicated that the existence of a gap is not a factor, this means that 53% (17 + 21 = 38) of the 72 program directors said that either they don’t consider gaps as a factor in their program’s interview and admission decisions or that gaps had no effect on those decisions.

**Figure 8:**
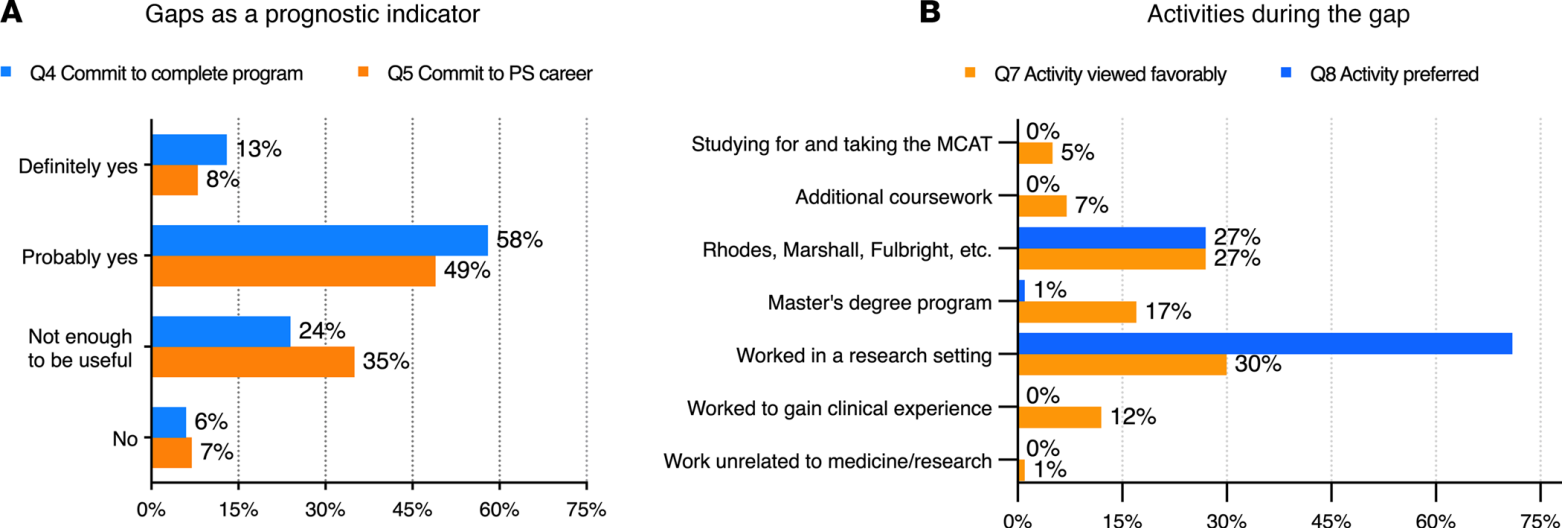
Program directors’ survey. Are gaps a prognostic factor, and what is the preferred activity during the gap? (**A**) Directors’ opinions regarding the extent to which an applicant choosing to take a gap indicates a commitment to complete the program (blue) and to pursue a career as a physician-scientist (red). Percentage of total responses is shown. (**B**) Directors’ choices from dropdown lists of preferred activity (red) and activity viewed most favorably (blue) (*n* = 71).

Program directors were then asked if they view a gap spent doing research as being a favorable prognostic indicator for either commitment to complete an MD-PhD program or commitment to a career as a physician-scientist (Figure 8A). 71% answered probably or definitely “yes” to the first part of the question; 58% answered probably or definitely “yes” to the second. Notably, however, a sizable fraction of the program directors answered either “not enough to be useful” (24% and 35%) or “no” (6% and 7%) to these same questions, which suggests that, in the very least, there is not a consensus on whether taking a gap to demonstrate commitment will accomplish that goal for applicants.

Despite the lack of unanimity about the use of gap information in decision making, all but one of the program directors indicated that they care how candidates spend their time during a gap between college and medical school. Those 71 directors were then asked about which activities they view favorably and which they most prefer (Figure 8B). Working in a research setting or completing a prestigious individual fellowship were by far the most highly preferred activities.

**Figure 9:**
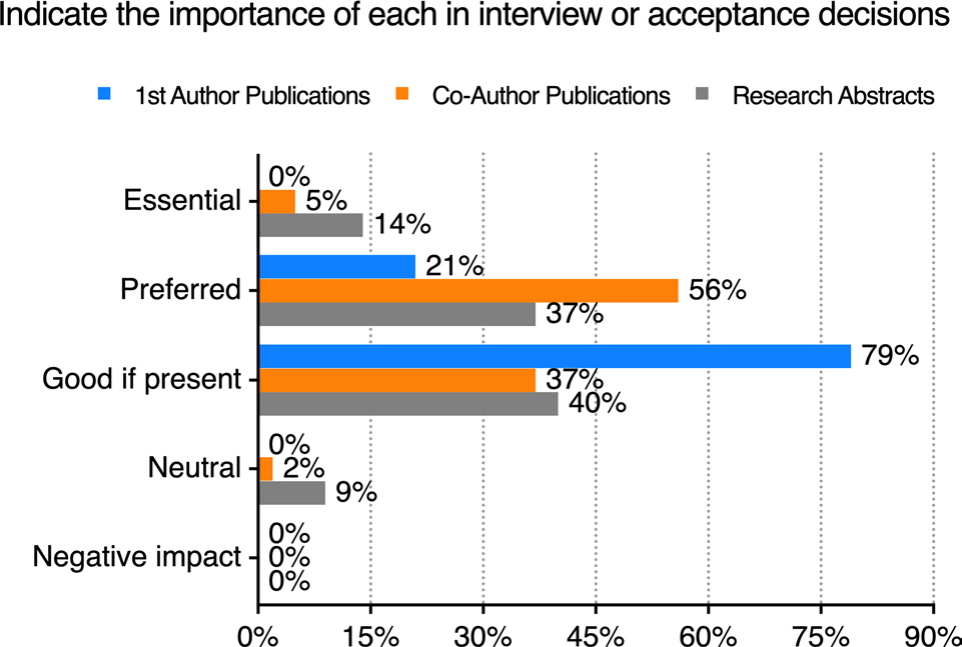
Program directors’ survey. The importance of an applicant having publications or abstracts. The directors’ survey asked whether secondary applications asked about publications. If they responded yes (*n* = 43 of 71), they were asked to rate the importance of first author papers (blue), coauthored papers (orange), and abstracts (gray). The percentage of responses is shown.

Finally, program directors were asked how they view prior publications at the time of application. 43 (60%) answered “yes” to the question “Do you ask applicants whether they already have research papers submitted, in press, or published in your program’s application?” Of those 43, none considered first author publications to be essential. All 43 said that first author publications were either preferred or good if present but not negative if absent (Figure 9). The corresponding replies for whether coauthor publications were considered essential, preferred, or good were 5%, 56% and 37%, respectively. Notably, a few of the programs that said they asked for publication-related information indicated that they were neutral (i.e., do not really care) about the answer.

### Does taking a gap shorten the time to degree?

**Figure 10:**
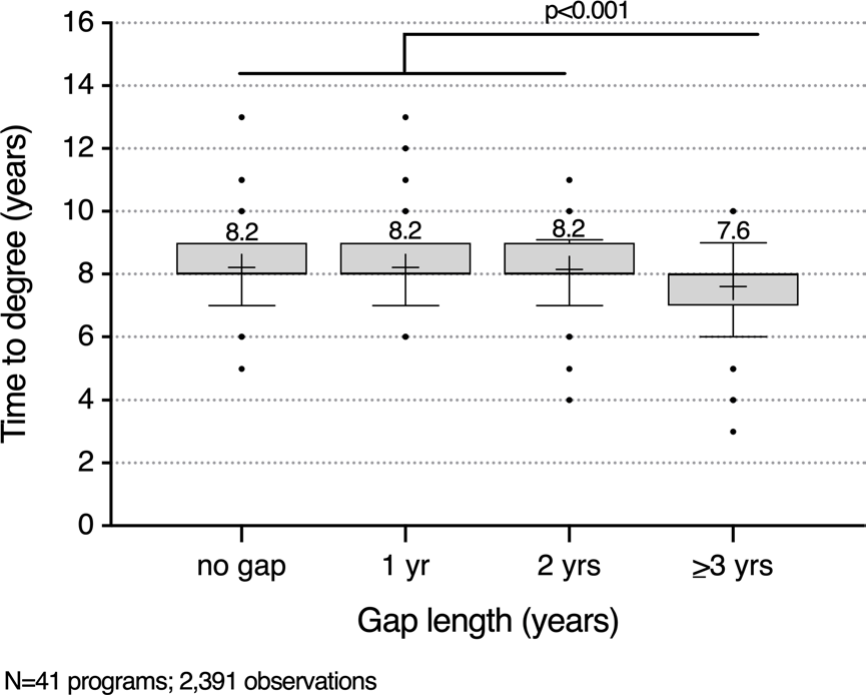
Relationship between gap duration and time to degree. Forty-one programs provided deidentified data on 2391 program graduates who entered training after 2006 and graduated by 2021, 1103 with no gap, 581 with a 1-year gap, 401 with a 2-year gap, and 306 with a gap of 3 or more years. Boxes indicate the 25th to 75th percentiles, and whiskers indicate the 10th and 90th percentiles. Points above and below the whiskers are shown. The “+” in each box is the mean; this value is shown above each box. The time to degree was the same for those who took either no gap after college or a gap lasting 1 or 2 years. The average time to degree for those with a gap of 3 or more years was approximately 0.6 years (7 months) shorter (*P* < 0.001 by 1-way ANOVA).

With most current students reporting that they took a gap before entering an MD-PhD program and that they typically used the time to work in a research setting, we were interested whether the additional research training and experience prior to matriculation facilitated faster completion of an MD-PhD program. Data on gap length and time to degree for matriculants entering on or after 2006 and graduating by 2021 was requested from all participating programs, of which 41 programs were able to provide information on 2391 graduates. Among the reported graduates, 1103 trainees did not take a gap and 1288 did. There was no difference in the average time to degree for those with gaps of 0, 1, or 2 years (Figure 10). The average time to degree for those with a gap of 3 or more years was approximately 0.6 years (7 months) shorter than for trainees who took a shorter gap or who entered training without a gap (*P* < 0.001 by 1-way ANOVA).

## Discussion

Recent events, including the COVID-19 pandemic, have highlighted the importance of biomedical research and the role that physician-scientists play in translating research from bench to bedside in a timely manner. Despite the importance of this career path, concerns have been raised for decades about the inadequate number of physician-scientists and the lack of necessary diversity in the physician-scientist workforce (3, 6, 16, 17). While MD-PhD programs are not the only path to becoming a physician-scientist, they have attracted considerable attention because of their ability to integrate research and clinical training and because many graduates of these programs have proven to be successful in sustaining research careers (2). At a few medical schools, MD-PhD students represent 20% or more of each entering class, but nationwide only 3% of medical students are enrolled in an MD-PhD program. With the exception of the 2021 admissions cycle, the number of applications to MD-PhD programs has remained flat, while medical school applications have increased.

A number of factors have been cited to explain this lack of growth in the applicant pool. Notable factors include the time required to complete an MD-PhD program, the opportunity costs of deferred employment, the perception that physician-scientists start their families late and few manage to achieve acceptable work/life balance, limits on the number of positions available for physician-scientists, and lower salaries compared with either full-time clinical practice or tech sector jobs. The training path for physician-scientists often begins before or during college and extends through medical school, residencies, clinical fellowships, and postdocs, especially for those headed for careers in academia. As a result, training to become a physician-scientists can begin at 18 and last until 40 years of age. This path used to be considerably shorter. Reasons for why it has grown include increases in both the time to degree in MD-PhD programs and the time to a first job after postgraduate clinical training is complete (2, 15).

Here, we have focused on a less appreciated contributor to the extension of training time — the increased prevalence of gaps taken between college and medical school. The data show that gap prevalence for MD-PhD students rose from 53% in 2013 to 75% in 2020, greatly outstripping the growth in gaps for MD students during the same time period. The dominant reason for this increase was applicants’ belief that gaps would increase their chances of being admitted, especially to the most competitive programs. Most trainees report using the gap period to build their research resumes. Notably, AAMC MSQ survey data show that research year(s) between college and medical school are common. However, the fraction of medical students doing gaps has now become substantially lower than the fraction of entering MD-PhD students (Figure 1A), and the reasons appear to be somewhat different. Many entering medical students cited a career shift as part of their decision to apply to medical school after gaining research experience. “Career switch” was not one of the prespecified choices in the current MD-PhD trainee survey, but respondents could write in additional reasons for deciding to do a gap, and 541 chose to do so. Only 16 (3%) described a career switch as a driving factor. That small group had an average gap duration nearly twice as long as those who didn’t describe a career switch (3.8 vs. 2.1 years).

Although large amounts of data were obtained from trainees, it is important to note as a potential limitation that only 71% of current trainees in participating programs chose to complete the survey. Because there is no way to know how the nonresponders would have answered the questions in the survey, we used AAMC data on applicants and matriculants to calculate gap prevalence for all successful and unsuccessful applicants to MD-PhD programs from 2013 to 2020. Those data confirm the rise in gap prevalence observed in our survey and show that unsuccessful applicants as a group were at least as likely to have taken a gap as those who were successful. This excludes the hypothesis that failure to gain admittance was due to a failure to take a gap. However, it does not exclude the possibility that at any given program there is a strong preference (conscious or unconscious) for applicants who have done gaps. The only way to determine the presence of a preference for a gap would be to evaluate program-specific data on those admitted versus those who were not admitted.

The opinions about gaps expressed by MD-PhD program directors proved to be more nuanced than the rush of applicants toward gaps would suggest. Only half (48%) indicated that they somewhat or strongly prefer applicants who have taken a gap, meaning that half of program directors do not. Although an even higher fraction (57%) felt that the decision to take a gap before applying probably or definitely forecasts commitment to a career as a physician-scientist, there are no data available to test this impression. We also found no evidence that time spent doing research in a gap shortens the time to degree, as might be expected if students arrive with greater skills and/or focus.

Why then is gap prevalence increasing? Part of the reason may be following the leader. Because most students in MD-PhD programs have done a research gap, it is not surprising that undergraduates will conclude that this is one way to increase their chances of admission. Compounding that impression is the advice that they get from college prehealth advisors, who often base their recommendations on past experience, and program directors, who emphasize the importance of meaningful, sustained research experiences. It seems that no matter the ambivalence that may be felt by individual program directors, collectively program directors are delivering the message that more research before applying to MD-PhD programs is better, full-time research is better than part time, and having one’s name on publications is desirable, although by no means essential. Advice about the value of gaps is also readily available via internet search engines. Some of it appears in forum discussions among those who have applied or are in the process of applying. Some of these opinions are from businesses selling their services to would-be physicians and physician-scientists.

A final factor that may be influencing admissions policies that favor gaps is the need to meet NIH expectations. NIGMS MSTP T32 grants currently support 50 MD-PhD programs, including, it should be noted, the 3 programs with which the authors of this study are affiliated. Over the past decade NIH expectations for data about applicants and matriculants has changed to require months of full-time research. Coincidentally or not, this change in reporting requirements coincided with the increase in gap prevalence.

What, then, is the harm if entrants into MD-PhD programs spend 1, 2, or 3 years doing full-time research before they dive into medical school? In addition to research and improved competitiveness, trainees in the present study listed a gap as a chance to take a break before an extended and intense training program, a wish to not spend their senior year applying, a desire to confirm their choice of a research-oriented career, and, in some cases, a need to reapply after falling short the first time. All of these reasons are perfectly understandable. Gaps are not without value in individual cases and, for some applicants, that value may be great enough to warrant taking the time. However, several adverse effects are also worth considering, including an extension of the already long time required before MD-PhD trainees can start their careers. Arguably, the greatest potential harm is when gaps result in a decision not to apply to MD-PhD programs, which may especially be the case for individuals from groups that are economically disadvantaged or UIM. Most MD-PhD programs provide tuition waivers and stipends for their students; however, the time invested in training comes with real costs. Training carries large opportunity costs for college graduates who could otherwise be headed toward careers that pay better sooner. For some without adequate family or personal resources, a gap and overall time in training can be a drain on financial resources that may not be fully compensated for by salaries earned many years later, and it may be limiting the diversity of applicants to MD-PhD programs.

At the end of the survey, trainees were invited to share any additional thoughts, and it is worth including a few of them here. Comments from the group that did not take a gap are especially informative. Although many reaffirmed their decision not to do a gap, others expressed regret, believing that they would have been more competitive, more mature, or more prepared if they had done so. Some expressed frustration that research gaps have become the new normal: “Gap years of research should not be a requirement for MD-PhD programs, that’s what the PhD is for.” One respondent reported, “I got feedback on my F30 [NIH fellowship application] that I could’ve had more publications if I’d taken a gap year, which seems like a ridiculous expectation given the length of the MD-PhD program already.” “I think the increasing number of students taking gap years is because of admission preference for the increased experience that comes with a gap year, which doesn’t necessarily reflect an individual’s capability to succeed in the program or future career.” These comments are anecdotal, but we agree with them.

Here is one comment that especially stood out: “The way selection is being done at the moment, you have essentially 90% of every matriculating class compos[ed] of students from research-intensive and/or ‘prestigious’ institutions. This exacerbates the inequities we already know of in undergraduate admissions and also causes the undesirable outcome where our MSTP trainees are not as diverse as can be.” The comment about 90% of matriculating MD-PhD students coming from research-intensive, prestigious institutions is not borne out by the data in this study; however, the comment that a perceived preference for applicants from research-intensive and/or prestigious institutes has an adverse effect on either racial or socioeconomic diversity is worrisome in an era when MD-PhD programs enroll relatively few students from groups considered to be UIM.

In conclusion, the present study shows that the majority of successful MD-PhD applicants are pausing after college to do more research and that they are doing so in part because of what they perceive to be a requirement for admission. That perception may not always be correct and may not be applicable to every program, but the survey data reported here make a compelling case. However, as tempting as it may be to believe, there are at present no data that would allow programs to conclude that gaps improve short- or long-term physician-scientist career outcomes. Unfortunately, the data set assembled in the National MD-PhD Outcomes study did not include college graduation year (2), which would have made it possible to calculate gap length. Absent such data, we suggest that policies and practices be revisited to assess their effect on applicants and societal needs for an active and diverse physician-scientist workforce. If a consensus emerges in the physician-scientist training community that undergraduates should hear that gaps are fine but not a requirement, then the answers to the survey question “How did you conclude that a gap was necessary?” suggest that applicants are getting the message from multiple sources — including college prehealth advisors, program directors, current MD-PhD students, and online forums — and integrating them amorphously into the most frequent answer “Personal opinion” (Tables 6 and 7). All of these sources will have to be addressed in as many settings as possible, but especially in outreach talks by program directors, on program websites, and in organized events.

## Methods

In January 2021 an email was sent to a listserv of MD-PhD programs maintained by the National Association of MD-PhD Programs. Program directors were invited to participate in a study of students’ reasons for taking a gap between college and matriculation into MD-PhD programs. 73 MD-PhD programs, including 49 of the 50 NIGMS MSTP-supported programs, agreed to participate. A Qualtrics survey link was sent to each program to distribute to their students. Survey links were tagged with a unique “source” string so that the number of responses from each participating program could be tracked. 3545 completed surveys were received (of a total 5007 students enrolled in the 73 participating institutions). One respondent provided an undergraduate graduation year and MD-PhD matriculation year that indicated a 1-year gap. This individual was excluded from the analysis. The overall response rate to this survey was 70.8%. For multiple response set questions where respondents could “select all that apply,” a subsequent question asked them to identify the primary reason from among those they had chosen.

Deidentified information on applicants and matriculants to MD-PhD programs for each year from 2010 to 2020 was obtained from the AAMC under a data licensing agreement. The information that was provided and used in the present study included the year of college graduation, the outcome of each application (accepted MD-PhD, accepted MD, rejected), and the year of matriculation into an MD-PhD program (from which gap length was calculated). For data on the percentage of matriculating medical students who took a gap, we obtained the AAMC MSQ All Schools Summary Reports for the period from 2013 through 2020 either from the AAMC website (https://www.aamc.org/data-reports/students-residents/report/matriculating-student-questionnaire-msq) or via a request to the AAMC Data Unit.

A follow-up survey of the directors of the participating MD-PhD programs was sent in April and May, 2021. 72 program directors provided data for an overall response rate of 97%. 41 program directors also answered a subsequent request by providing deidentified information on college graduation year, MD-PhD program matriculation year, and MD-PhD graduation year on 2391 program graduates who matriculated in 2006 or after. Gap duration was calculated as year of MD-PhD matriculation minus year of college graduation. Time to degree was calculated as year of MD-PhD graduation minus year of matriculation. We recognize that leaves of absence might alter calculated time to degree but we did not request information on leaves of absence.

The student survey asked all respondents demographic questions regarding race/ethnicity, gender, year of college graduation and MD-PhD program matriculation, college major, questions about the extent of undergraduate research experience, and number of publications at the time of MD-PhD program application. Race and ethnicity choices were consistent with NIH guidelines described in NOT-OD-15-089 (18); respondents could select all that applied. Respondents could optionally identify their undergraduate institution. Students who had 1 or more years between undergraduate graduation and MD-PhD program matriculation (gap years) were asked multiple-choice and open-ended questions focused on what students did during their gap years, why they chose to take a gap, and their research experiences prior to matriculation. All respondents were provided with an open-ended final question: “Please share any additional thoughts or comments regarding research requirements or gaps taken prior to matriculation in your MD-PhD program.” The survey is provided in Supplemental Methods. Data were analyzed using descriptive statistics and graphs. Responses were anonymized using the Qualtrics “anonymize response” feature and source identification was removed prior to analysis. No analysis was performed on a program-by-program basis. Responses from each program’s students will be returned to the program director in a fully deidentified format. The trainee survey was reviewed and granted exempted status by the University of Pennsylvania IRB.

For the purposes of this analysis, we defined UIM to include those individuals who self-identified with one or more of the following groups: Black or African American, Hispanic or Latino, American Indian or Alaska Native, and Native Hawaiian or Pacific Islander. Because of the limited numbers of American Indian, Alaska Native, and Native Hawaiian or Pacific Islander trainees in MD-PhD programs and the increasing number of individuals who self-identify as members of more than one group, we chose in most of this analysis to report UIM as a single group, breaking out individual groups only when we felt that sufficient information was available. For some analyses, we defined program size based on the number of reported students. “MSTP funded” included those programs funded by NIGMS T32 grants during the July 1, 2020 to June 30, 2021 fiscal year. Undergraduate majors were categorized as physical sciences (chemistry, computer science, engineering, mathematics, physics), biological sciences (biological and biomedical sciences, health sciences), and social sciences (humanities, social sciences, psychology).

### Statistics.

Where indicated in the text and figures, group comparisons were made using a 1-way ANOVA with Tukey’s HSD post hoc test for multiple comparisons after confirming that the data satisfy the assumption of a normal distribution. A *P* value of less than 0.05 was considered significant.

### Study approval.

The surveys were reviewed by the University of Pennsylvania IRB and deemed to meet eligibility criteria for IRB review exemption, authorized by 45 CFR 46.104, category 2.

## Author contributions

LFB, RMF, and MHA collected data, analyzed data, and wrote the manuscript.

## Supplementary Material

Supplemental figure 1

## Figures and Tables

**Table 7:**
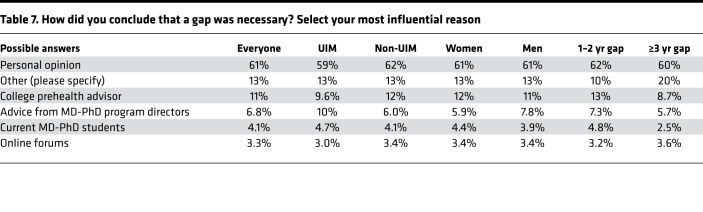
How did you conclude that a gap was necessary? Select your most influential reason

**Table 6:**
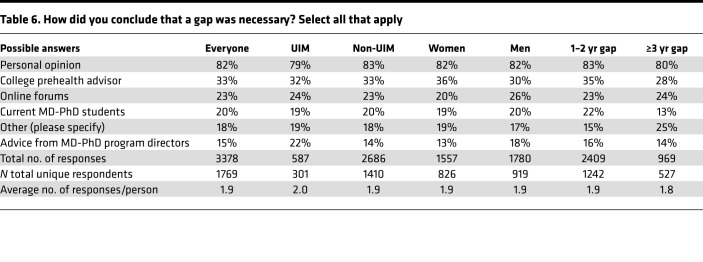
How did you conclude that a gap was necessary? Select all that apply

**Table 5:**
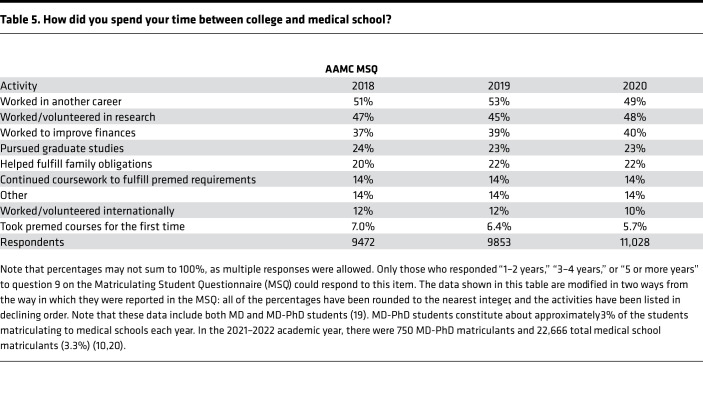
How did you spend your time between college and medical school?

**Table 4:**
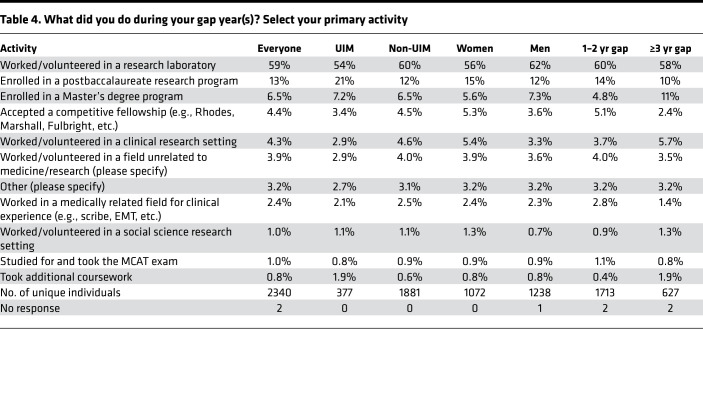
What did you do during your gap year(s)? Select your primary activity

**Table 3:**
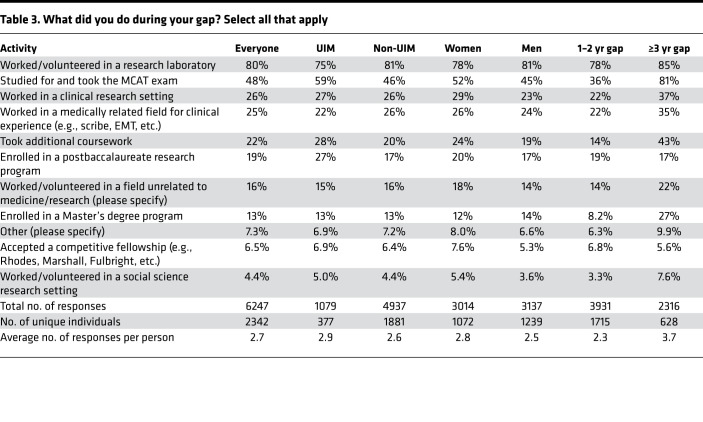
What did you do during your gap? Select all that apply

**Table 2:**
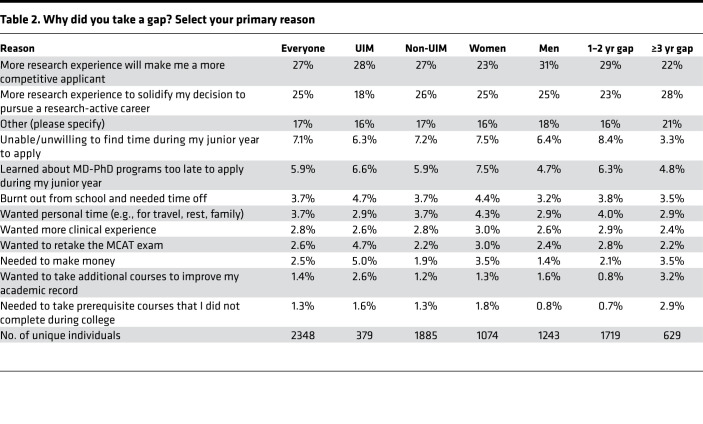
Why did you take a gap? Select your primary reason

**Table 1:**
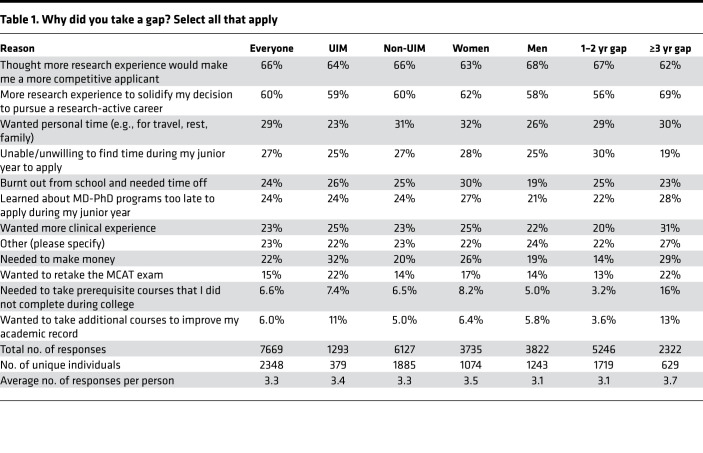
Why did you take a gap? Select all that apply

## Version 1 03/22/2022

Electronic publication
