# Supplementary figures and images for: Gaps between college and starting an MD-PhD program are adding years to physician-scientist training time
Source: JCI Insight. 2022 Mar 22;7(6):e156168. doi: 10.1172/jci.insight.156168 (PMC8986071; doi:10.1172/jci.insight.156168)

# Gap prevalence (top 30)

Number of alumni who completed the survey

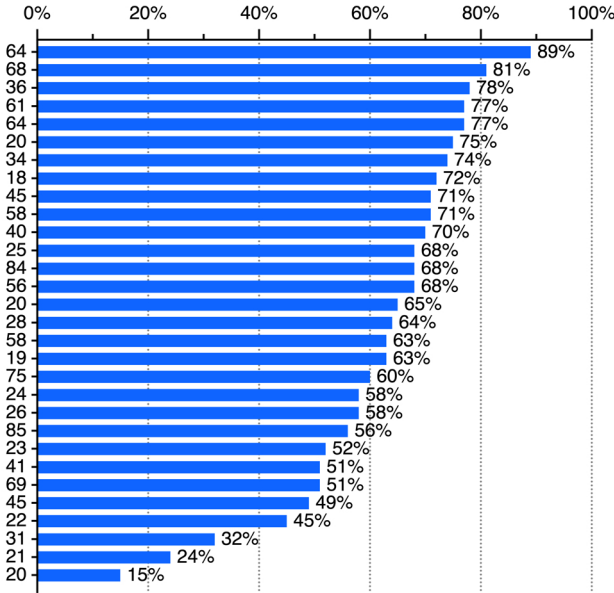

Supplement: Supplemental figure 1 [file jciinsight-7-156168-s114.pdf]
